# Hazard function analysis of prognosis after recurrent colorectal cancer

**DOI:** 10.1007/s00423-024-03308-w

**Published:** 2024-04-13

**Authors:** Ichiro Ise, Kazushige Kawai, Daisuke Nakano, Misato Takao, Soichiro Natsume, Hiroki Kato, Sakiko Nakamori, Akira Dejima, Tatsuro Yamaguchi

**Affiliations:** Department of Colorectal Surgery, Tokyo Metropolitan Cancer and Infectious Diseases Center, Komagome Hospital, 3-18-22 Honkomagome, Bunkyo-ku, Tokyo, 113-8677 Japan

**Keywords:** Colorectal cancer, Recurrence, Hazard function, Prognosis

## Abstract

**Background and objectives:**

Mean survival time (MST) is used as the indicator of prognosis in patients with a colorectal cancer (CRC) recurrence. The present study aimed to visualize the changes in death risk after a CRC recurrence using hazard function analysis (HFA) to provide an alternative prognostic indicator to MST.

**Methods:**

The medical records of 725 consecutive patients with a recurrence following R0 radical surgery for CRC were retrospectively reviewed.

**Results:**

The five-year, post-recurrence survival rate was 37.8%, and the MST was 3.5 years while the risk of death peaked at 2.9 years post-recurrence. Seven variables were found to predict short-term survival, including the number of metastatic organs ≥ 2, non-surgical treatment for the recurrence, and a short interval before recurrence. In patients with a recurrence in one organ, the MST was four years, the peak time of death predicted by HFA was 2.9 years, and the five-year survival rate was 45.8%. In patients with a surgical resection of the recurrence, the MST was 8 years, the peak time of death was 3.3 years, and the five-year survival rate was 62%.

**Conclusions:**

The present study established a novel method of assessing changes in mortality risk over time using HFA in patients with a CRC recurrence.

## Introduction

Colorectal cancer is one of the most common, malignant diseases and the second most frequent cause of cancer-related death worldwide [1]. The cumulative recurrence rate is reportedly around 20% [2], and surgical resection is the standard treatment if the recurrence is curatively resectable. In cases where surgical treatment is unsuitable, systemic chemotherapy may be considered; owing to recent advances in chemotherapy, the median survival time (MST) post-recurrence has lengthened to more than 30 months [3]. However, despite these treatments, a large proportion of patients with colorectal cancer recurrence still die from the disease. Therefore, it is important to predict the survival outcome whenever a recurrence is observed. The Kaplan–Meier survival estimate is the standard method of assessing survival outcomes in clinical studies [4], and the median survival time (MST), or the period during which 50% of patients in a given population die according to the Kaplan–Meier estimate, is generally used as the standard index of patient prognosis [5]. However, in the daily clinical setting, the MST should not be considered an ideal prognostic indicator because a patient with a recurrence really wants to know two things: “What is the probability of my survival?” and “If I can’t survive the recurrence, how long can I survive?” The second question can be paraphrased as, “When does my risk of death peak?” The answers to these questions cannot be found in the MST alone; rather, the focus needs to be placed on two survival indicators, the five-year survival probability and an assessment of changes in the hazard rate of death over time using hazard function analysis (HFA). HFA, a method of analysis proposed by Simes and Zelen, plots data with the horizontal axis representing time and the vertical axis representing the hazard rate [6]. HFA enables the risk of an event, such as death or the recurrence peak [4, 6], to be visualized. Cancer conditions at the time of a recurrence vary widely between individuals from a surgically resectable, single recurrence in the liver to multiple, unresectable metastases in multiple organs. The resulting prognosis accordingly varies widely. The present study aimed to demonstrate improved prediction of the prognosis by calculating the five-year survival probability and the most frequent time of death after recurrence in each of the patients enrolled.

## Methods

### Patients

The medical records of 725 consecutive patients with a CRC recurrence who had undergone surgery with curative intent between January 2005 and December 2016 in the Department of Surgery at Tokyo Metropolitan Cancer and Infectious Diseases Center at Komagome Hospital were retrospectively reviewed. The right-sided colon was defined as the bowel proximal to the splenic flexure consisting of the cecum and the ascending and transverse colon, and the left-sided colon was defined as the bowel distal to the splenic flexure consisting of the descending and sigmoid colon and rectum. Tumor staging was performed using the TNM classification of malignant tumors, 8th edition [7]. All the patients had undergone postoperative surveillance according to the surveillance protocol defined by the Japanese Society for Cancer of the Colon and Rectum guidelines [2]. Tumor markers (carcinoembryonic antigen, carbohydrate antigen 19–9) were examined every three months, computed tomography (CT) was performed every six months, and a total colonoscopy was recommended after the first postoperative year and every two to three years thereafter.

A recurrence was diagnosed on the basis of biopsy, MRI, PET/CT or CT findings at follow-up. The recurrence date was recorded as the date of diagnosis of the first recurrence. The present study was approved by the institutional review board of the study center (reference number: 3080).

### Statistical analysis

Variables with *P* < 0.05 on univariate analysis were subjected to multivariate Cox proportional hazards analysis. The five-year cumulative incidence of recurrence was calculated using the Kaplan–Meier method. Survival estimate was made using JMP Pro 16.2 (SAS Institute Inc., Cary, NC, USA), and hazard function analysis was performed using R 3.6.1 with the muhaz package (R Project for Statistical Computing, Vienna, Austria; http://www.r-project.org/).

## Results

**Fig. 1:**
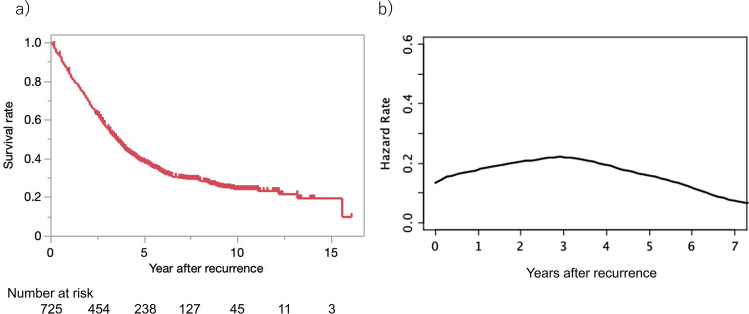
Survival plots after the recurrence of colorectal cancer. **a** Kaplan–Meier curve for survival after recurrence. **b** Hazard function plot representing changes in risk of death after recurrence

Table 1 shows the general characteristics of the patients included in this study. The most frequent site of the initial cancer was the rectum (54.9%). Adjuvant chemotherapy was administered in 43% of the patients. The most frequent site of recurrence was the liver (37.8%) followed by the lung (31.9%) and lymph node (23.2%). The median interval between the initial surgery and the recurrence was 1.0 year, and the median follow-up period from the recurrence was 3.4 years. The five-year survival rate after a recurrence was 37.8%, and the MST was 3.5 years (Fig. 1a). The risk of death peaked at 2.9 years post-recurrence (Fig. 1b).

**Table 1:** Patient characteristics

Variable		*n* (%)
Number of patients		725
Sex (male)		438 (60.4%)
Age (years)		65 (17—91) †
Primary tumor related variables		
Cancer site ††	Right-sided colon cancer	157 (21.7%)
	Left-sided colon cancer	170 (23.4%)
	Rectal cancer	398 (54.9%)
T stage ††	Tis—1	23 (3.2%)
	2	60 (8.3%)
	3	392 (54.1%)
	4	250 (34.5%)
*N* stage ††	0	237 (32.7%)
	1	276 (38.1%)
	2	212 (29.2%)
M stage	0	605 (83.4%)
	1	120 (16.6%)
Lymphatic invasion		480 (66.1%)
Venous invasion		648 (89.2%)
Well or moderately differentiated adenocarcinoma		654 (90.0%)
Adjuvant chemotherapy	None	413 (57.0%)
	Present without oxaliplatin	278 (38.3%)
	Present with oxaliplatin	34 (4.7%)
Variables at time of recurrence		
Metastatic organ	Liver	274 (37.8%)
	Lung	231 (31.9%)
	Peritoneum	81 (11.2%)
	Lymph node	168 (23.2%)
	Local	115 (15.9%)
	Other	67 (9.2%)
Number of metastatic organs	1	558 (77.0%)
	2	135 (18.6%)
	3	26 (3.6%)
	4	6 (0.8%)
Treatment for recurrence	Chemotherapy	272 (37.5%)
	Surgery	351 (48.4%)
	Best supportive care	60 (8.3%)
	Other	42 (5.8%)
Interval between initial surgery and recurrence (years)		1.0 (0.09 – 11.7) †
Follow-up period from recurrence (years)		3.4 (0.2—16.1) †

^†^ Data are presented as the median (range)

^††^ For cases of multiple lesions, the site or stage of the most advanced lesion is given

Table 2 shows the results of univariate and multivariate analyses of the association between variables and post-recurrence survival. Based on the results, the following seven variables were found to predict shorter survival: age ≥ 60 years, T4, N ≥ 1, histological type other than well or moderately differentiated adenocarcinoma, number of metastatic organs ≥ 2, non-surgical treatment for the recurrence, and interval between initial surgery and the recurrence < 2 years.

**Table 2:** Multivariate analysis of survival after recurrence

		Univariate analysis	Multivariate analysis		
		*P*-value	Hazard Ratio	95% CI	*P*-value
Sex	Male vs. female	0.1137			
Age (years)	< 60 vs. ≥ 60	0.0019	1.62	1.33–1.98	< 0.0001
Primary tumor related variables					
Cancer site	Right sided vs. ≥ left sided	0.1787			
T stage	Tis/1/2/3 vs. T4	< 0.0001	1.41	1.18–1.70	0.0002
*N* stage	N0 vs. N ≥ 1	< 0.0001	1.42	1.16–1.75	0.0008
M stage	M0 vs. M1	0.9378			
Lymphatic invasion	Absent vs. present	0.0007	1.19	0.97–1.47	0.0982
Venous invasion	Absent vs. present	0.2803			
Histological type	Well or moderately differentiated adenocarcinoma vs. other histological types	0.0026	1.65	1.25–2.19	0.0004
Adjuvant chemotherapy	Absent vs. present	0.7465			
Recurrence-related variables					
Number of metastatic organs	1 vs. 2 or more	< 0.0001	1.77	1.45–2.18	< 0.0001
Treatment for recurrence	Surgical resection vs. other treatment	< 0.0001	3.64	2.97–4.46	< 0.0001
Interval between initial surgery and recurrence	< 2 years vs. ≥ 2 years	0.0133	0.7	0.56–0.87	0.0015

*CI* Confidence interval

**Fig. 2:**
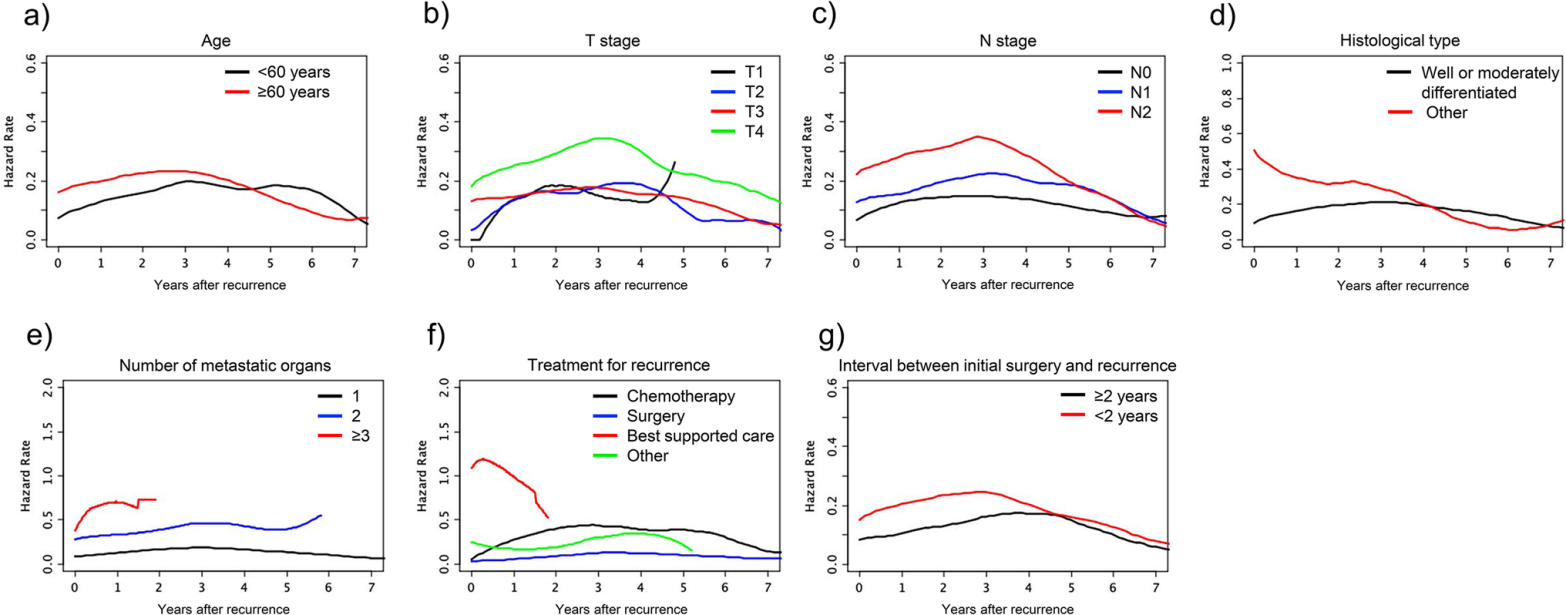
Differences in risk of death by prognostic factor. Hazard function plots stratified by (**a**) age (black line, < 60 years old; red line, ≥ 60 years old), **b** T stage (black line, T1; blue line, T2; red line, T3; green line, T4), **c** N stage (black line, N0; blue line, N1; red line, N2), **d** histological type (black line, well or moderately differentiated adenocarcinoma; red line, others), **e** number of metastatic organs (black line, 1; blue line, 2; red line, ≥ 3), **f** treatment for recurrence (black line, chemotherapy; blue line, surgery; red line, best-supported care; green line, others), and (**g**) interval between initial surgery and recurrence (black line, ≥ 2 years; red line, < 2 years)

Changes in the hazard rate of death with the passage of time in the seven variables were compared (Fig. 2). The older patient group had a shorter peak time (2.79 years) than the younger group (3.03 years) (Fig. 2a). The hazard rate was higher in T4 patients (0.342) than in T1/2/3 patients (0.183, 0.194, 0.178, respectively), peaking at 3.11 years after recurrence (Fig. 2b). The hazard rate was higher in N2 patients (0.350) than in N0/1 patients (0.147, 0.226), peaking at 2.86 years (Fig. 2c). Histological types other than well or moderately differentiated adenocarcinoma were associated with a greater risk of death, which was especially high in the early period after the detection of a recurrence (Fig. 2d). The hazard rate increased as the number of metastatic organs increased. If a metastasis was found in only one organ, the peak hazard rate was 0.185; if a metastasis was found in two organs, it increased to 0.465. For three or more organs, the hazard rate increased to 0.702 (Fig. 2e). The hazard rate was lower in patients with surgery for the initial recurrence (0.130) than in those with chemotherapy, best supportive care, etc. (0.443, 1.186, and 0.350, respectively) (Fig. 2f). The hazard rate was higher (0.245) if the interval between surgery and recurrence was < 2 years than if it was longer (0.174) and peaked at 2.85 years (Fig. 2g).

The MST, five-year survival after recurrence, and peak time of death were compared according to the number of metastatic organs and treatment for the recurrence (Table 3). If the recurrence was confined one organ, the MST was four years whereas the peak time of death was 2.9 years, and 45.8% of the patients survived five years post-recurrence. The MST of the patients with surgery for a recurrence was eight years, their peak time of death was 3.3 years, and 62% survived five years post-recurrence (Table 3).

**Table 3:** Survival outcome by the number of metastatic organs or treatment

		MST (year)	5-year survival (%)	Peak hazard rate	Peak time (year)
Number of metastatic organs	1	4.2	45.8	0.185	2.9
	2	2.0	13.7	0.465	3.2
	≥ 3	0.9	0	0.702	0.95
Treatment for recurrence	Chemotherapy	2.3	16.3	0.443	2.9
	Surgery	8	62.0	0.130	3.3
	Best supportive care	0.5	2.6	1.186	0.3
	Other	3.1	26.0	0.350	3.8

*MST* Median survival time

## Discussion

The present study is the first to investigate survival after a colorectal cancer recurrence using HFA. A previous study reported that the incidence of recurrence by organ was 35.5%, 23.8%, and 19.9% for the liver, lung, and local organ, respectively [8], which was in line with the present findings.

The patients’ question, “How long can I survive?” should be a combination of two questions, “How likely am I to die from cancer?” and “If I am to die, when?”. The answer to the former question should be the 5-year survival, and the answer to the latter question should be the peak time determined by hazard function analysis. Figure  1 shows that around 60% of the patients died within five years of a recurrence, and HFA found a peak time of three years, indicating that death after the diagnosis of a recurrence occurred most frequently around three years post-recurrence.

Seven factors were found to be independent variables of survival post-recurrence: age, T stage, N stage, histological type, number of metastatic organs, treatment, and the interval between the initial surgery and the recurrence. Among these, treatment and the number of metastatic organs were the most important as indicated by their high hazard ratio. Several studies have demonstrated that right-sided colon cancer, histological type other than well or moderately differentiated adenocarcinoma, lymph node metastasis, presence of peritoneal metastasis at the time of recurrence, recurrence in ≥ 2 organs, surgically unresectable recurrence, and recurrence within postoperative 2 years [9, 10] were indicative of shorter survival after a colorectal cancer recurrence, in line with the findings of the present study.

Although old age and a short interval until recurrence were both risk factors of poor survival, it is unclear whether the death rate was high or the survival time was short. In the present study, HFA demonstrated that the death risk was higher during the first three years after a recurrence in the elderly patient group and the shorter interval group but converged thereafter (Fig. 2a, g). This may be explained by the less intensive treatment given to the elderly patients because of the presence of comorbidities and their impaired performance status [11, 12] and by rapid tumor growth in the shorter interval group [13–15]. Ryuk et al. reported that the five-year survival rate of colorectal cancer patients with a recurrence within two years after primary tumor resection was 34.7% while in patients with a longer interval it was 78.8%, corroborating our findings [14].

A higher stage of the primary tumor was also associated with poor prognosis post-recurrence. The hazard rate remained higher in T4 than T1 to T3 and in N2 than in N0 or N1 throughout the entire period probably because of higher invasiveness and metastatic potential (Fig. 2b, c). The peak time in all the groups was around three years, with the difference being non-significant. Connell et al. reported that the stage of the primary tumor was also an independent prognostic factor of tumor recurrence [16] as seen in the present study.

Patients with a histological type other than well or moderately differentiated adenocarcinoma mostly had poorly differentiated or mucinous adenocarcinoma and extremely short, post-recurrence survival (Fig. 2d). Their hazard rate peaked markedly within one year post-recurrence, suggesting that most patients with a recurrence of one of these histological types died within one year post-recurrence despite the frequency of the histological types being only around 10% [17]. Among the histological types in question, poorly differentiated adenocarcinoma is known to be especially malignant owing to an accumulation of genetic alterations and higher proliferative potential [18, 19] and also is reportedly less sensitive to chemotherapy than other types [20, 21].

As the number of metastatic organs increased, the hazard rate also increased possibly because R0 resection tended to be unfeasible in cases of multiple organ metastasis, and also because carcinomas with metastases to more than one organ may be more aggressive (Fig. 2e). Previous studies have also reported that the number of metastatic organs is also an important prognostic factor [9, 22].

The hazard ratio was higher in patients receiving chemotherapy than in those with recurrence resection throughout the whole period, with the ratio plateauing from two to five years post-recurrence without a clear peak. This suggests that most patients were able to survive about two years with chemotherapy alone but began dying during the following three years, a finding which conforms closely with clinical experience.

Table 3 shows that the MST should not be treated as a reliable indicator of survival. For instance, in patients with a recurrence in only one organ, the five-year survival rate is 45.8%, i.e., nearly half the patients survive after a recurrence. However, death in the remaining half most frequently occurred at around three years post-recurrence, a much shorter period than the MST (four years). In contrast, patients with two metastases had a peak time of 3.2 years (for their hazard ratio), over one year longer than the MST (two years). Therefore, the combination of the five-year survival rate as assessed by the Kaplan–Meier curve and the peak time as assessed by HFA is closer to being the optimal prognostic indicator. HFA was also found to be quite useful in visualizing changes in death risk over time. Although HFA is widely used to analyze the occurrence of events not only in malignant diseases but also in benign diseases, such as surgical site infections [23, 24], the present study demonstrated its applicability to the survival analysis of recurrent colorectal cancer.

The present study has several limitations. First, the study cohort included old cases that lacked a sufficient pre- and post-recurrence surveillance period. Therefore, the survival outcome might have been better if the patients had received current, cytotoxic and molecularly targeted drugs. Second, this study was conducted at a single center, and a multicentric cohort study is needed to verify its findings.

In conclusion, we developed a novel method of assessing changes in mortality risk over time in patients with a postoperative recurrence of colorectal cancer using HFA. The findings of this study will hopefully be of use to physicians and patients alike in daily clinical practice.

## Data Availability

The data that support the findings of this study are available on request from the corresponding author. The data are not publicly available due to privacy or ethical restrictions.
